# Efficacy of Suppression of Serum Transthyretin With Patisiran and Vutrisiran in Variant ATTR Amyloidosis: An Observational Crossover Study

**DOI:** 10.1161/CIRCULATIONAHA.125.076330

**Published:** 2026-02-03

**Authors:** Yousuf Razvi, Muhammad Umaid Rauf, Aldostefano Porcari, Josephine Mansell, Awais Sheikh, Adam Ioannou, Carol J. Whelan, Lucia Venneri, Ana Martinez-Naharro, David F. Hutt, Dorota Rowczenio, Janet A. Gilbertson, Ashutosh D. Wechalekar, Helen J. Lachmann, Philip N. Hawkins, Marianna Fontana, Julian D. Gillmore

**Affiliations:** National Amyloidosis Centre, University College London, Royal Free Hospital, London, United Kingdom.

**Keywords:** amyloid, cardiomyopathies, heart failure, prealbumin, RNA interference, transthyretin

Transthyretin amyloidosis (ATTR) is caused by deposition of amyloid derived from serum transthyretin (sTTR). Long-term experience in light chain amyloidosis and amyloid A amyloidosis has confirmed that greater suppression of circulating amyloid fibril precursor proteins results in better outcomes.^1,2^ Although currently unproven, this paradigm is anticipated to hold true in ATTR.

Patisiran and vutrisiran are small RNA interference agents that reduce sTTR concentration ([sTTR]) by silencing hepatic TTR mRNA, each meeting primary end points in phase 3 trials of ATTR cardiomyopathy and ATTR polyneuropathy.^3,4^ We present data comparing [sTTR] in a single-center ATTR cohort treated sequentially with both drugs.

TRANSCEND (Transthyretin Amyloidosis: Neuropathy, Senility, Cardiomyopathy, Evaluation, Natural History and Diagnosis) is a long-term observational study of patients with ATTR amyloidosis attending the United Kingdom National Amyloidosis Centre. We studied patients who received patisiran 3-weekly between 2016 and 2023 and subsequently switched to 3-monthly vutrisiran upon United Kingdom availability. All patients had serial [sTTR] measurements pre patisiran, post patisiran, and post vutrisiran; each sample was obtained >28 days post treatment initiation. Median values of multiple [sTTR] measurements were used for analysis. Change in [sTTR] was expressed as percentage of the pretreatment [sTTR] and as absolute [sTTR] post treatment. There was no pause between patisiran therapy completion and commencement of vutrisiran such that the prepatisiran [sTTR] was used as the baseline concentration for both treatments. Patients who received TTR stabilizers or other gene silencers were excluded from the study. The lower limit of detection of the sTTR assay was 6 mg/L; values below this were assigned a concentration of 5 mg/L.

The study received United Kingdom Health Research Authority approval (IRAS-ID 256590, REC-ID 21/PR/0620), and all participants provided consent for publication of their anonymized data, which can be made available upon reasonable request.

We studied 112 patients with variant ATTR polyneuropathy (women:men 39:73; mean [SD] age 60.5 [19.8] years; 69 [62%], 18 [16%], 15 [13%], and 10 [9%] had polyneuropathy disability stage I, II, IIIa, and IIIb, respectively), 95 (85%) of whom had concomitant ATTR cardiomyopathy (70 [74%], 22 [23%], and 3 [3%] National Amyloidosis Centre stage I, II, and III, respectively). In total, 214 samples across a median (interquartile range) follow-up of 16.8 (17) months and 194 samples across a median (interquartile range) follow-up of 5.9 (5.6) months were studied following patisiran and vutrisiran administration, respectively. sTTR was measured using Thermo Fisher Scientific binding site transthyretin assays, measured on the Optilite analyzer. Mean (SD) pretreatment [sTTR] was 210 mg/L (80); men had higher [sTTR] than women (220 versus 180 mg/L, *P*=0.01), consistent with published data.^5^ The mean (SD) median postpatisiran [sTTR] was 38 (41) mg/L, representing an 80% reduction from baseline. The mean (SD) median postvutrisiran [sTTR] was 13 (18) mg/L, representing a 93% reduction from baseline. Paired *t* tests demonstrated that absolute and percentage sTTR reductions from baseline were significantly greater with follow-on vutrisiran therapy (both *P*<0.001; Figure 1). In a linear mixed-effects model with random intercept per patient, adjusting for time since dosing, mean [sTTR] was significantly lower following vutrisiran compared with patisiran therapy (difference, –27 mg/L; *P*<0.001). An [sTTR] <6 mg/L occurred in 28 of 214 (13.1%) postpatisiran and 114 of 194 (58.8%) postvutrisiran samples (χ²=91.6, *P*<0.001). Accordingly, 101 of 112 (90%) patients achieved lower [sTTR] with follow-on vutrisiran than patisiran alone, with 11 patients showing the converse. The median (interquartile range) time between dosing and first posttherapy [sTTR] measurement was 11.7 (8.6) versus 5.6 (0.7) months (*P*<0.001) for patisiran and vutrisiran respectively; at these early time points, mean (SD) posttreatment [sTTR] was significantly lower following vutrisiran than patisiran (12.5 [±17.7] versus 40.5 [±40.7] mg/L, *P*<0.001). To exclude declining nutritional status as a contributor to the lower [sTTR] on vutrisiran, we compared the last postpatisiran with the first postvutrisiran [sTTR] (median interval, 12.5 months); mean (SD) additional sTTR reduction on vutrisiran was 26 (45) mg/L (33%), consistent with an additional drug effect. While percentage sTTR reduction was greater in men than women, there was no significant difference in absolute posttreatment [sTTR] between sexes, reflecting the higher pretreatment [sTTR] in men.

**Figure 1.**
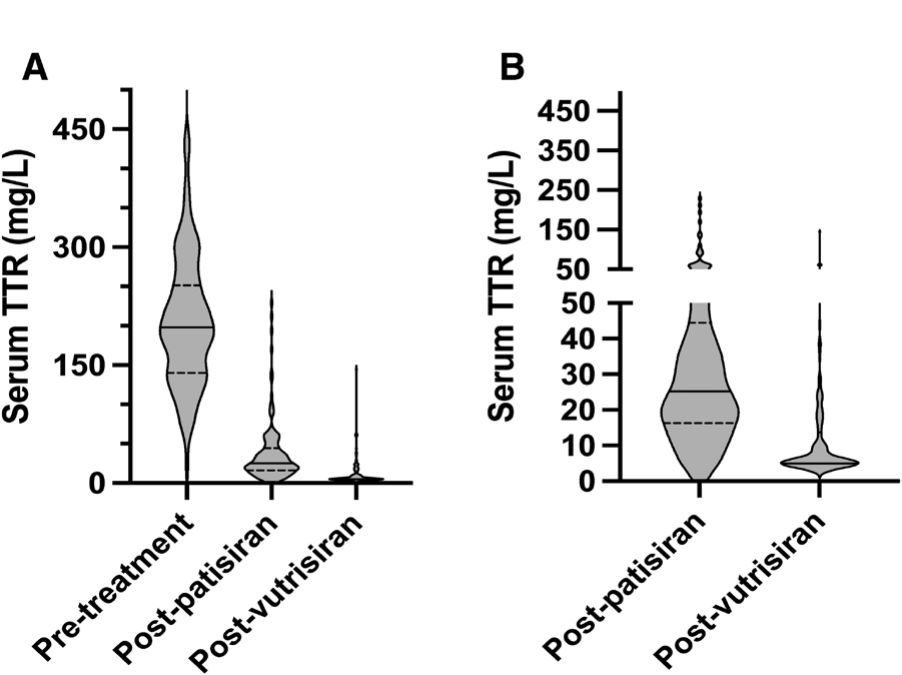
**Violin plots demonstrating the magnitude of reduction of serum transthyretin (TTR) in response to patisiran and vutrisiran among 112 patients with variant TTR amyloidosis (41 [36%] T60A, 24 [21%] V30M, 11 [10%] V122I, 7 [6%] S77Y, 30 [27%] rarer variants**) **who received both agents. A**, The distribution of pretreatment, postpatisiran, and postvutrisiran TTR levels. **B**, The residual postpatisiran and postvutrisiran TTR concentration. In all modes of analysis, vutrisiran appeared to be associated with statistically significantly greater TTR reduction than patisiran (*P*<0.001). The solid bars denote the median, and interrupted bars denote the first and third quartiles.

This study in a single-center clinical cohort, which used patients as their own crossover controls, suggests that vutrisiran may achieve greater sTTR suppression than patisiran; 90% of patients had further sTTR reduction following a switch to vutrisiran. Vutrisiran therapy appeared to be associated with lower [sTTR] compared with patisiran across multiple time points, including at a median of 5.6 months after its initiation, at the expected nadir of its pharmacodynamic effect.

Our study has limitations; it is retrospective, requiring prospective validation.. Patients were not permitted to recover to baseline sTTR before switching from patisiran to vutrisiran. We were unable to evaluate change in [sTTR] following a switch from vutrisiran to patisiran, since this sequence did not arise clinically. Since the lower limit of detection of the sTTR assay was 6 mg/L, and values below this were assigned 5 mg/L, true suppression may have been modestly underestimated. While similar findings may be expected in wild-type ATTR cardiomyopathy, this group was not studied.

In summary, vutrisiran achieved a mean 93% reduction in [sTTR] compared with an 80% reduction during prior patisiran treatment in this diverse United Kingdom variant ATTR polyneuropathy cohort. Ongoing studies will relate the magnitude of TTR suppression to outcomes. These findings suggest that N-acetylgalactosamine-conjugated small RNA interference agents may offer a superior real-world approach for hepatic *TTR* mRNA silencing and future therapeutic development.

## ARTICLE INFORMATION

### Sources of Funding

This study was supported by funding for TRANSCEND from Alnylam Pharmaceuticals.

### Disclosures

Dr Razvi reports grant income from AstraZeneca, consulting fees from BridgeBio, and speaker fees from Bayer. Prof Fontana is supported by a British Heart Foundation Intermediate Clinical Research Fellowship (FS/18/21/33447). Prof Fontana reports participation in consultancy/advisory boards for Alnylam, Alexion/ Caelum Biosciences, AstraZeneca, Bridgbio/Eidos, Prothena, Attralus, Intellia Therapeutics, Ionis Pharmaceuticals, Cardior, Lexeo Therapeutics, Janssen Pharmaceuticals, Prothena, Pfizer, Novo Nordisk, Bayer, and Mycardium. Prof Julian Gillmore reports participation in consultancy/advisory boards for Alnylam, Astrazeneca, ATTRalus, Eidos, Intellia Therapeutics, and Ionis Pharmaceuticals, Pfizer.
